# Current Data Regarding the Relationship between Type 2 Diabetes Mellitus and Cardiovascular Risk Factors

**DOI:** 10.3390/diagnostics10050314

**Published:** 2020-05-16

**Authors:** Cosmin Mihai Vesa, Loredana Popa, Amorin Remus Popa, Marius Rus, Andreea Atena Zaha, Simona Bungau, Delia Mirela Tit, Raluca Anca Corb Aron, Dana Carmen Zaha

**Affiliations:** 1 Department of Preclinical Disciplines, Faculty of Medicine and Pharmacy, University of Oradea, 410073 Oradea, Romania; v_cosmin_15@yahoo.com (C.M.V.); raluca14@yahoo.com (R.A.C.A.); danaczaha@gmail.com (D.C.Z.); 2 Department II of Internal Medicine, Clinical County Emergency Hospital of Oradea, 410169 Oradea; Romania; popa_lori2000@yahoo.com (L.P.); popa_amorin@yahoo.com (A.R.P.); rusmariusr@yahoo.com (M.R.); 3 Department of Medical Disciplines, Faculty of Medicine and Pharmacy, University of Oradea, 410073 Oradea, Romania; 4 Faculty of Medicine, “Iuliu Hațieganu” University of Medicine and Pharmacy, 400000 Cluj Napoca, Romania; andreeaatenazaha@gmail.com; 5 Department of Pharmacy, Faculty of Medicine and Pharmacy, University of Oradea, 410028 Oradea, Romania; mirela_tit@yahoo.com

**Keywords:** diabetes, cardiovascular risk, pathogenic mechanisms, SGLT-2 inhibitors, GLP-1 agonists

## Abstract

Reducing cardiovascular risk (CVR) is the main focus of diabetes mellitus (DM) management nowadays. Complex pathogenic mechanisms that are the subject of this review lead to early and severe atherosclerosis in DM patients. Although it is not a cardiovascular disease equivalent at the moment of diagnosis, DM subjects are affected by numerous cardiovascular complications, such as acute coronary syndrome, stroke, or peripheral artery disease, as the disease duration increases. Therefore, early therapeutic intervention is mandatory and recent guidelines focus on intensive CVR factor management: hyperglycaemia, hypertension, and dyslipidaemia. Most important, the appearance of oral or injectable antidiabetic medication such as SGLT-2 inhibitors or GLP-1 agonists has proven that an antidiabetic drug not only reduces glycaemia, but also reduces CVR by complex mechanisms. A profound understanding of intimate mechanisms that generate atherosclerosis in DM and ways to inhibit or delay them are of the utmost importance in a society where cardiovascular morbidity and mortality are predominant.

## 1. Introduction

Cardiovascular complications account for more than 70% of all hospital admissions in diabetic patients in the USA [1]. The macrovascular complications include coronary artery disease (CAD), cerebrovascular disease and peripheral artery disease. In diabetic patients, the acute myocardial infarction risk is 2.13 times greater for men and 2.95 times greater for women compared to the respective non-diabetic populations [2]. Numerous studies present conflicting results regarding the impact of gender on cerebrovascular disease—several argue for a greater risk in women and others in men. Nonetheless, the general population presents a 2–4-fold increase in CAD risk for diabetic patients in comparison with the non-diabetic ones [3]. Moreover, cardiovascular risk (CVR) factors in diabetic patients have a substantial role in the overall risk as 75–80% of diabetic patients suffer from hypertension, 70–80% present high LDL-cholesterol levels, and 60–70% are clinically obese. For a long-time, it has been considered that diabetes mellitus (DM) represents a counterpart of cardiovascular disease; however, this has remained debatable.

The CAD risk was equal for patients with a history of over 10 years progression of DM and those with cardiovascular disease [4]. Furthermore, diabetics present higher mortality rates after acute events, i.e., for these patients, the post-infarction mortality rates are 40% greater in comparison with non-diabetic patients [5].

DM is an independent risk factor for stroke. Moreover, diabetic patients present a certain stroke pattern, they suffer more frequently of ischaemic rather than haemorrhagic strokes and the lacunar strokes represent a majority [6]. The prevalence of peripheral artery disease in diabetics is approximately 20%. This is clinically presented as intermittent claudication in one in three of patients and is asymptomatic for the rest of them. Screening is performed through the ankle-brachial index whereby values <0.90 signify positive test results confirming the presence of this disease. Patients with peripheral artery disease present a high risk of developing lower limb ulcerations and 15 times greater risk of amputation in contrast with non-diabetic patients [7] as a result of infection with multiple pathogens [8].

In this review, the authors’ objectives were as follows: to identify comorbidities of DM patients that share the same pathogenic substrate with DM (insulin resistance and factors that further increased the atherosclerotic risk); to highlight the most common CVR factors in DM and the importance of identifying them early in a patient with diabetes, as well as their management according to the new guidelines. All these objectives were achieved by analysing the information provided by some of the most recent works published in the specialized literature.

## 2. Common Comorbidities in DM that Share the Same Substrate: Insulin Resistance and Further Increase of Cardiovascular Risk

### 2.1. Dyslipidaemia

Dyslipidaemia is frequently found in up to 70–80% of diabetic patients [3]. It is defined by quantitative as well as qualitative serum lipids alterations, the most common being triglyceride increase and HDL-cholesterol decrease. There are also numerous changes regarding LDL and very low-density lipoprotein (VLDL)-cholesterol particles [2].

Triglycerides increase is caused by the classic lipoprotein lipase inhibition in insulin resistance as well as the inhibition of hepatic lipase. Hormone-sensible lipase exhibits increased activity levels which generate an increase in free fatty acids levels that arrive to the liver. Insulin resistance increases hepatic synthesis of apolipoprotein B (apoB) and triglyceride rich VLDL particles [3]. It also reduces lipoprotein lipase activity which alters chylomicrons catabolism leading to an increase in triglycerides [2]. In type 2 DM, circulating VLDL particles are large VLDL1 particles rich in cholesterol and triglycerides exhibiting strong atherogenic features with high affinity for macrophages which turn them into foamy cells through phagocytosis [2].

Triglyceride-rich VLDL-cholesterol particles transfer triglycerides to the LDL and HDL-cholesterol particles [4]. Normally, HDL-cholesterol takes up cholesterol from various tissues being esterified by lecithin cholesterol acyltransferase. If the blood becomes rich in high VLDL levels, HDL exchanges its cholesterol for triglycerides through the cholesterol ester transfer-protein enzyme. Fast disposal of the triglycerides contained by these particles through the hepatic lipase leads to low levels of HDL-cholesterol in diabetic patients [4]. The same enzyme, cholesterol ester transfer-protein, exchanges cholesterol from LDL particles for triglycerides in VLDL particles. Increased metabolism of these triglycerides by the hepatic lipase leads to the genesis of small and dense LDL-cholesterol particles which are responsible for the atherogenesis in DM [5]. The latest research shows that a high triglyceride/HDL-cholesterol ratio is correlated with insulin resistance in type 2 DM and represents a cardio-vascular risk predictor [6].

### 2.2. Metabolic Syndrome

Metabolic syndrome (MS) significantly increases the risk of developing DM and CAD [7]. It is estimated that patients with MS have a five-fold greater risk of developing DM and a two-fold greater risk for CAD in comparison with patients without MS [7]. Studies show that 80% of all DM patients also suffer from MS in the USA [8]. Different organisations use different definitions of the metabolic syndrome.

World Health Organization defines MS creating one compulsory criteria: the presence of modified basal glycaemia, glucose tolerance alteration or DM + 2 or more of the following: increased arterial blood pressure values (>140/90 mm Hg), hypertriglyceridemia (>150 mg/dL), and/or low HDL-cholesterol (<35 mg/dL in men, <40 mg/dL in women) and microalbuminuria (albuminuria ≥20 μg/min or urine albumin/creatinine ratio ≥30 mg/g) [9].

The International Diabetes Federation (IDF) defines MS as central obesity (abdominal circumference ≥94 cm in men and ≥80 cm in women) as well as two of the following components: baseline glycaemia >100 mg/dL, triglycerides >150 mg/dL, HDL-cholesterol <40 mg/dL in men, <50 mg/dL in women, arterial hypertension (SBP >130 mmHg or DBP >85 mm Hg) [9]. Among MS components, obesity has been reported to be the most important predictive factor [10]. Cardio-vascular risk is greater in MS than the cumulative risk of all its components [11] although there are studies that contradict this result [12]. Insulin resistance is the common key component which explains most of these issues as obesity determines insulin resistance, at first only in adipose tissues, then in the liver as well, due to the high release of free fatty acids which get intercepted by the liver, the ending result being fat metabolism alterations [13]. Besides its frequent correlation with DM, MS also associates with non-alcoholic steatohepatitis, polycystic ovary disease, sleep apnoea syndrome, hypogonadism and microvascular disease [13]. A method to prevent MS onset is weight loss of at least 5%–10% body weight in the case of obesity [13].

### 2.3. Non-Alcoholic Steatohepatitis

**Figure 1:**
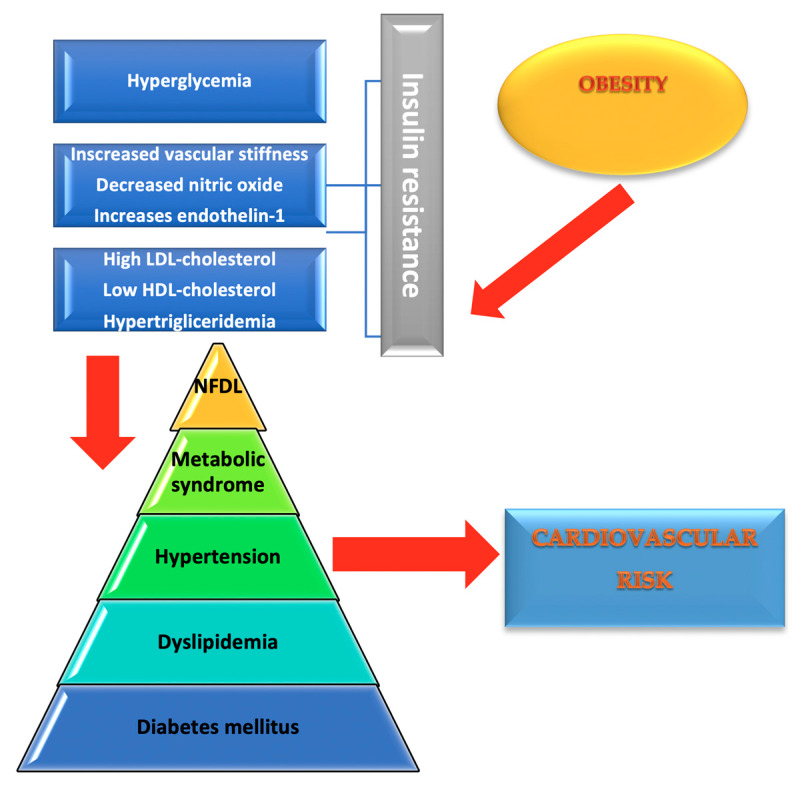
Pathophysiological alterations in obesity and diabetes mellitus.

Non-alcoholic steatohepatitis is commonly found in type 2 DM patients, its prevalence being around 70% [14]. Obesity causes insulin resistance which leads to free fatty acids build-up in the liver, increasing triglyceride synthesis. Type 2 DM favours hepatic steatosis progression through increased production of hepatic glucose which, together with the free fatty acids, provides the basis for triglyceride synthesis [14]. Steatosis in itself represents a risk factor for developing type 2 DM, inasmuch as to double it. Generally, all hepatic diseases cause changes in glucose metabolism; hepatic insulin resistance overstresses the pancreatic B cells. Increased hepatic fats build-up and their insufficient export leads to disease progression, thus converting non-alcoholic hepatic steatosis to steatohepatitis due to the toxic properties of fats [15]. Excessive lipids are broken down in the mitochondria increasing hepatic ROS levels and leading to the migration of macrophages and T lymphocytes to the hepatocytes which induces a significant local inflammatory response and increases proinflammatory cytokine levels [15]. As in the case of diabetics with MS, for patients with non-alcoholic hepatic steatosis weight loss and regular exercise can be beneficial as hepatic fat reduction and improvement of glycaemic control methods [15]. The pathophysiological alterations in obesity and DM are presented in Figure 1.

### 2.4. Chronic Kidney Disease (CKD)

CKD represents an independent risk factor for cardiovascular morbidity [16]. CKD prevalence in T2DM patients is over 50% [17]. All the risk factors that trigger and promote the progression of CKD in DM patients are caused by the presence of insulin resistance, which itself causes hypertension, dyslipidaemia, endothelial dysfunction and inflammation [18]. Early CKD develops insulin resistance and promotes the progression to more advanced stages by mechanisms such as sodium retention, activation of the sympathetic nervous system and decrease of the natriuretic peptide system synthesis [19]. Insulin resistance also mediates and accentuates the impact of CKD presence on the severity of cardiovascular disease. Not only that T2DM patients develop left ventricular hypertrophy because of hypervolemia that appears as a consequence of CKD, but also insulin resistance itself can trigger the expression of ectonucleotide pyrophosphatase phosphodiesterase 1 [ENPP1] gene, a gene known to be responsible of cardiac myocyte hypertrophy [20]. Higher HOAM-IR levels have been associated with higher risk of cardiovascular mortality in patients with peritoneal dialysis [21]. Focusing on non-pharmacological and pharmacological therapies that act on inhibiting most insulin-resistance generated risk factors (such as GLP-1 RA or SGLT-2 inhibitors) is an important step in minimalizing the impact of insulin resistance in T2DM patients in order to protect them against progression towards CKD [22,23].

## 3. Cardiovascular Risk Factors in DM—General Picture

DM itself represents a major CVR factor, the majority of diabetic patients’ deaths being due to cardiovascular complications [24]. The risk is further increased by the frequent association between obesity, hypertension and dyslipidaemia.

There are several CVR factors classifications for diabetics. One of these distinguishes two categories: the glycaemic factors and non-glycaemic ones: arterial hypertension, dyslipidaemia, obesity, smoking, chronic inflammation and microalbuminuria. Another classification mentions traditional (old age, male gender, hypertension, DM, dyslipidaemia, smoking, sedentary lifestyle, and familial history of CVD) and non-traditional risk factors. The non-traditional risk factors have been the subject of increasing research, although their specific impact on CVR has been difficult to assess—some examples include: insulin resistance, endothelial dysfunction (due to excessive vasoconstriction and reduced vasodilation), inflammation (high C reactive protein levels, high leukocytes), microalbuminuria, intima-media thickness, and coronary calcium score [25,26].

Hyperglycaemia is another CVR factor and its control is being highly debated. The UKPDS has shown that for patients with excellent glycaemic control, with a mean HbA1c <7%, it was observed a 16% reduction in cardiovascular complications in comparison with patients with mean HbA1c values of 7.9%, although this reduction was not statistically significant [27]. Similarly, the Action to Control Cardiovascular Risk in Diabetes (ACCORD) study has highlighted the same statistically insignificant reduction of cardiovascular events in patients with more intensive glycaemic control; moreover, this group of patients experienced more frequent hypoglycaemic events and weight gains of over 10 kg. The study was discontinued due to significantly high mortality in this group of patients [28]. The ADVANCE study compared intensive treatment with standard treatment for patients, five years after therapy initiation. Mean HbA1c was 6.5% in the first group and 7.3% in the second one. The intensive treatment group presented a lower microvascular complications incidence than the standard group, especially nephropathy, but optimal glycaemic control did not have impact on macrovascular complications [29]. These results support the hypothesis that hyperglycaemia is not the only responsible for increased CVR in diabetic patients: high BP values, dyslipidaemia, and non-traditional risk factors also being responsible and requiring multiple target therapy to reduce CVR.

Other studies have shown that prompt intensive hyperglycaemia treatment reduces CVR in patients without other risk factors. DCCT study highlighted a 47% risk decrease for any CVD and a 57% reduction of MI, stroke or cardiovascular-related risks causing death [30]. The characteristic of this study was that more intensive therapy initiation was done for young patients with type 1 DM without any cardiovascular history. Another study, carried out for recently diagnosed T2DM patients who received more intensive treatment, showed 15% MI risk reduction in patients receiving sulphonylurea or insulin and a 33% reduction in those receiving metformin [31]. These data led to the conclusion that more intensive therapy in DM is effective in CVR reduction when there are patients with no or little CV risks [27]. As far as glycaemic control is concerned, ADA recommends an optimal HbA1c value <7% [28]. ADVANCE and ACCORD studies have proven that very intensive hyperglycaemia treatment does not offer cardiovascular benefits for patients with arterial or cardiac disease history, nor for those with long-standing DM history, however more recent studies have highlighted the existence of new oral antidiabetics which significantly reduce the CVR in patients with CVD history [27,32]. Therefore, the oral antidiabetic choice seems to be more important than glycaemic control; these are usually added to the metformin monotherapy. The EMPA-REG study has shown the efficiency of empagliflozin/metformin, a SGLT-2 inhibitor, in decreasing CV mortality for diabetic patients with CV history with up to 38% [33]. The CANVAS study has proven that canagliflozin was also efficient in reducing CVR [34]. Also, liraglutide, a GLP-1 analogue, has been shown to be effective in reducing CVD in patients with long-term DM [35]. Metformin has proven cardioprotective effects, reducing the risk of cardiovascular mortality by 33% [31]. Metformin improves lipid parameters, causes a slight weight loss or impedes weight gain, lowers TAS and reduces oxidative stress and chronic inflammation [36,37,38]. The American Diabetes Association (ADA) recommends for patients with metformin therapy and lifestyle modification, who have a cardiovascular history, the addition of an oral antidiabetic drug, strongly evidenced to provide cardiovascular protection [37].

Arterial hypertension is one of the most important CVR factors in diabetic patients. Indeed, 77–87% of these subjects suffered from it [36,39]. ADA recommendations include target values of <140/90 mmHg but stricter limits should be considered in high risk patients: <130/80 mmHg or <120/80 mmHg [37]. However, a meta-analysis has shown that systolic values under 140 significantly reduce CVR but further decreasing it under 130 does not offer additional benefits. All antihypertensive drugs are efficient in reducing CVR among both non-diabetic and diabetic patients, but the latter particularly benefit from angiotensin converting enzyme inhibitors and angiotensin receptors blockers [38].

ADA recommends lifestyle changes for diabetic patients with values >120/80 mm Hg as they can reduce blood pressure values as well as support glycaemic control [40]. These changes include as follows: low salt intake (<2.3 g/day), 8–10 portions of fruits and vegetables every day, 2–3 portions of low-fat dairy products consumption, smoking cessation, and increasing physical activity [41]. Diabetics with values <160/100 mm Hg should be prescribed one antihypertensive drug belonging to the following groups: angiotensin-converting-enzyme (ACE) inhibitors, angiotensin receptor blockers (ARBs), diuretics (thiazide-like), dihydropyridine calcium channel blockers [41]. Naturally, these are added to the lifestyle changing measures. Patients with both diabetes and CKD should be treated with ACE inhibitors and ARBs [37]. However, these should never be given concomitantly due to risks of acute renal injury and hyperkalaemia. Patients who have values >160/100 mm Hg require the prescription of two different antihypertensive drugs. In cases of CKD, one of these drugs must be an ACE inhibitor or ARB. Regardless of hypertension values, if the target values of <140/80 mm Hg are not reached, one additional drug will be prescribed (ACE inhibitor/ARB/calcium channel blocker/diuretic). If the target is still not reached by using one diuretic, one calcium channel blocker, and one ACE inhibitor or ARB, the prescription of loop diuretics is recommended [37].

Another CVR factor in diabetic patients is dyslipidaemia. Decreasing LDL-cholesterol may reduce CVR by 20–50%. These patients mainly present small and dense LDL particles which easily traverse the arterial wall transforming into oxidized LDL due to the effects of oxygen reactive species [42]. The intake of statins may reduce LDL levels as well as CVR in diabetic patients. In primary prevention, it has been shown that even low doses of statins are effective in reducing cardiovascular events risk by 37% [43]. The importance of LDL-cholesterol reduction is proven by the findings that demonstrate that each mmol/L decreases CVR by 21% [44]. ADA recommends the use of medium-dose statins for diabetics without cardiovascular history and high-dose statins for those with cardiovascular history. The therapeutic target for the former is LDL <100 mg/dL and <70 mg/dL for the latter [44]. Recently, there has been interest in researching the effect of triglycerides increase and HDL-cholesterol decrease. Evidence suggests that hypertriglyceridemia leads to an increase in potential atherogenic triglyceride rich VLDL1 particles [2]. Fibrates are effective in reducing triglyceride levels and increasing HDL-cholesterol levels, thus reducing CVR [45]. The ACCORD-LIPID study has found a reduction of CVR by 7% in diabetic patients who were prescribed fibrates in addition to simvastatin, however not statistically significant [46]. The FIELD study indicated HDL-cholesterol growth by 5% and triglyceride reduction by 37% in diabetic patients on fibrate treatment. The non-fatal myocardial infarction was reduced by 24% and the cardiovascular mortality risk suffered an insignificant reduction [47]. Further research within the FIELD study has proven fibrates to be beneficial in significantly reducing CVR by 27% in patients who presented levels of triglycerides ≥240 mg/dL and HDL-cholesterol <40 mg/dL (men), <50 mg/dL (women) [47]. High triglycerides and low HDL-cholesterol are a frequent association in DM. The triglyceride/HDL ratio has proven to be a CVR predictive factor; when its value is >4 it represents an extremely high risk of cardiovascular events [48]. In addition, this ratio correlates with the LDL-cholesterol type, therefore a high ratio is associated with type B particles—small, dense and intensely thermogenic [49].

Recent literature data regarding T2DM patients considered that non-HDL cholesterol level measurements associated with LDL-C/HDL-C ratio could be used as markers of dyslipidaemia [50]. Non-HDL cholesterol is an equivalent of the total quantity of lipoprotein containing apolipoprotein B (apo B) [51]. This protein has a proatherogenic effect, therefore the determination of non-HDL cholesterol has been validated as a useful marker for the risk of cardiovascular disease in current guidelines [52]. Liu et al. demonstrated that an increase of non-HDL cholesterol by 1mg/dL is associated with an increase of cardiovascular mortality with 5% among patients with T2DM [3]. In their study [53], the value of non-HDL cholesterol was 1.5–2.5 higher among patients with diabetes compared with non-diabetic patients. Numerous studies promote the idea that non-HDL cholesterol has a better predicting accuracy for cardiovascular disease than other lipid fractions much more explored in studies, such as LDL-cholesterol and triglycerides [54,55]. Non-HDL cholesterol is also a strong predictor of metabolic syndrome, because non-HDL cholesterol is mostly the sum of VLDL particles with high triglyceride content and other apo B containing particles. Hypertriglyceridemia is a consequence of insulin resistance. Therefore, high triglyceride levels lead to high VLDL-synthesis, and a global increase in non-HDL cholesterol [56]. Non-HDL cholesterol determination is also simpler and more convenient than the determination of LDL-cholesterol and can be performed without fasting, from random serum sample [50]. In patients with DM that generally have numerous comorbidities, a target of non-HDL cholesterol <100mg/dL can be attained by an adequate therapy with statins, ezetimibe and, when needed, fenofibrate and omega 3 fatty-acids supplementation [57]. Data from NHANES study demonstrated that over a period of 17 years, among individuals with atherosclerotic disease, non-HDL cholesterol decreased by 21% as statin usage rose from 37% in the 1999–2000 period to 69% in 2015–2016 [58], confirming the efficacy of statin treatment in reducing non-HDL cholesterol. Recent data present the serum non-HDL cholesterol level as an efficient biomarker of coronary heart disease in patients with CKD. Regular evaluation of serum non-HDL-C levels may present clinical relevance for the efficient prophylaxis of cardiovascular incidence for patients with CKD that present increased risk of CVD [59,60].

DM leads to high activation and aggregation of thrombocytes that is a CVR factor. Primary prevention of cardiovascular disease with aspirin in diabetics remains controversial and is currently indicated only in secondary prevention [61]. The recommended dose is 75–162 mg/day [37]. Patients with a recent history of acute coronary syndrome must be prescribed double anti-aggregation therapy with aspirin and clopidogrel for one year.

### 3.1. Prediction of Cardiovascular Risk in the Diabetic Patient Based on Risk Equations

CVR prediction is important in patients with DM in identifying high-risk patients and choosing the therapeutic strategy. DM represents a CVD factor, considered by some authors to be a CVD equivalent and, in the diabetic patient, the presence of other CVD factors varies from one patient to another, thus leading to different categories of CVD. Each CVR factor present in the diabetic patient, such as hypertension or dyslipidaemia, influences the CVD and it is necessary to apply scores that provide data as close to reality as possible on the CVR by combining the impact that each factor has. There are several risk scores, some of them being specific for patients with diabetes because they take into account the glycaemic parameters while others are more suitable for the general population as they do not take glycaemic parameters into account.

Framingham and SCORE risk scores are some of the most commonly used CVR prediction scores in the general population. Within these scores DM is only a factor of CVD, the duration of the disease and the glycaemic control not being taken into account. The Framingham score predicts CVR over the next 10 years and includes the following variables: sex, age, total cholesterol and HDL-cholesterol, systolic blood pressure, blood pressure treatment, smoking status, and the presence of DM [47]. A score below 10% is considered low, a score between 10–20% is considered intermediate, a score above 20% is considered high. The SCORE project score considers the patient’s sex, age, SBP values, cholesterol value and smoking status as variables [62]. A score above 10% is considered very high, a score between 5% and 10% high, a score between 1% and 5% moderate, and a score below 1% is considered low.

The UKPDS risk engine predicts CVR in the diabetic patient, taking into account HbA1c values, DM duration and other CVR factors. Numerous studies have compared CVR scores in terms of risk prediction accuracy. The results are often contradictory. Some studies indicate that both the UKPDS and Framingham scores accurately identify patients with high CVR, but both scores overestimate the risk [63]. Comparing CVR predicted by UKPDS risk engine, Framingham score, and JALS-ACC, UKPDS risk engine had the highest accuracy in predicting CVR [64]. Other studies give different results, i.e., the Framingham score and the UKPDS score overestimate the CVR. However, both had the ability to identify patients with high CVR [65]. Data from the meta-analyses show that diabetes specific CVR scores, such as UKPDS or ADVANCE, appear to have a slight advantage over scores designed for the general population [66,67].

The assessment of CVR in the diabetic patient is particularly important for identifying patients in the high and moderate risk category and for initiating the multifactorial treatment of hyperglycaemia and other risk factors such as hypertension or dyslipidaemia. In newly diagnosed patients, by calculating CVR through the UKPDS risk engine, the category of high-risk subjects had the greatest benefit from reducing CVR, being prescribed drugs with cardioprotective effect. The lowest benefit was for patients registered at low risk category [67]. These data demonstrate the importance of scores in therapeutic decision making in patients newly diagnosed with DM; however, there remains the risk that less attention will be paid to multifactorial treatment in these subjects.

Different studies have identified risk categories for diabetic patients with low and high CVR. The categories of patients with high CVR were represented by the elderly, males, smokers and those with low socioeconomic status [68]. Some studies have determined CVR in patients newly diagnosed with DM by the UKPDS risk engine. The diagnosis of CVD in diabetic patients had an impact on the therapeutic decision. In a study on newly diagnosed diabetic patients, using a value of 20% to define high CVR, 20.9% of patients fell into this category by calculating the Framingham score and 21.7% fell into this category by using the UKPDS risk engine [69]. Statin treatment in patients over 45 years of age has proven to be cost effective in reducing CVD in newly diagnosed patients. It seems that in the newly diagnosed patients the intensive glycaemic control significantly reduces CVD. Thus, the risk of mortality through myocardial infarction was 15% lower in patients with sulphonyl urea or insulin treatment compared to those who were only recommended lifestyle changes and 39% lower in patients treated with metformin than those to whom only lifestyle changes were recommended [31,70].

Therefore, the evaluation of CVD in the diabetic patient is especially important at the time of diagnosis, as this is the best therapeutic window for long term reduction of CVD, numerous studies proving that after the onset of cardiovascular complications, glycaemic supervision no longer has a significant impact on primary prevention but having an important role in the control of the risk factors. The newly diagnosed diabetic patient, without cardiovascular complications, benefits the most from the multifactorial therapeutic intervention.

### 3.2. Modern Management of Cardiovascular Risk Factors in DM

#### 3.2.1. Glycaemic Target and Managing Hyperglycaemia

As far as blood glucose levels recommendations go, ADA 2017 and ADA 2018 advise aiming for HbA1c <7%. This analysis should be done at least twice/year in patients reaching the target and every 3 months in those who have difficulties reaching it or with changes in their therapeutic regime. In newly diagnosed patients it should be aimed for fasting glucose between 80 and 130 mg/dL and post-prandial glucose <180 mg/dL [32,37].

The first therapeutic step in hyperglycaemia includes lifestyle changes and Metformin. This can be prescribed unless otherwise contraindicated and if HbA1c values are <9%. Patients with higher values than this should be promptly put on dual therapy and those with HbA1c ≥10% should benefit from insulin therapy [37].

Lifestyle changes include diet and increasing physical activity. Diabetics are recommended to consume whole grains, vegetables, fruits, low fat dairy products, lean meat, nuts and seeds. Obese patients should lose at least 5% body weight as this provides better glycaemic and risk factors control. At least 150 min of moderate-to-high intensity physical activity per week are recommended. Smoking cessation and psychosocial support are also very important for diabetic patients [37].

Metformin remains an extremely important antidiabetic in T2DM treatment because it has multiple advantages. Firstly, it is an oral drug which offers cardiovascular protection. One study has compared the effect of metformin vs. sulfonylureas or insulin treatments on a 10-year period; the first group reported a 33% decrease in acute myocardial infarction risk while the latter a 15% decrease [31]. Other studies confirmed these results by proving that patients undergoing coronarography while on metformin treatment had a 69% lower risk of acute myocardial infarction than those on insulin therapy [71]. Weight gain is not a side effect of metformin but, on the contrary, metformin provides a slight weight loss [72], has anti-inflammatory benefits [73,74], reduces oxidative stress [75,76], lowers endothelial dysfunction [69], improves lipid parameters by reducing triglycerides and LDL-cholesterol [72], and reduces hypertension [77,78].

After three months of metformin treatment and lifestyle changes, for the patients who have not reached their glycaemic goals a second oral antidiabetic should be added. Studies have shown that any oral antidiabetic drug added to the initial treatment reduces HbA1c levels by almost 1% [79]. The main difference between ADA 2017 and 2018 guides is choosing the second antidiabetic in patients with atherosclerotic disease [32,37]. Thus, the patients without CVD can benefit from any of the following drug classes: sulfonylureas, DPP-4 inhibitors, GLP-1 agonists, thiazolidinediones and basal insulin. According to ADA 2018, atherosclerosis patients should benefit from drugs which offer cardiovascular protection: SGLT-2 inhibitors or GLP-1 agonists. In case the goal is not reached within 3 months, another drug belonging to a different class is added. Mixed injectable treatment is recommended if the goal could not be reached with three drugs after another three months [37].

Among the new categories of pharmaceutical formulations used in the therapy of diabetes, GLP-1RA and SGLT2-I are encouraging alternatives. In the treatment of T2DM, SGLT2 inhibitors represent the latest therapeutic category accepted. Their action is to supress, in the proximal convoluted tubule of the kidney, the SGLT2 transport proteins. As these transporters represent almost 90% of the total resorption of filtered glucose in the body, they are valuable instruments in controlling the blood glucose. Being linked to decreases of 0.5–1% in HbA1c, SGLT2 inhibitors represent efficient alternative therapy choices for T2DM [80].

Besides their efficiency in treating diabetes, SGLT2 inhibitors are also helpful in weight loss as well as in the treatment of macrovascular and microvascular complications associated with T2DM [33,81,82]. Furthermore, SGLT2 inhibitors revealed favourable results in treating CV diseases. Moreover, SGLT-2 administration is correlated with renal protective effects; it is known that in patients with DM, CKD is highly prevalent mostly because of the association of hyper-glycemia, dyslipidaemia and high blood pressure [83]. The decrease in sodium reabsorption in the proximal renal tubule leads to a higher concentration of sodium at the level of macula densa, which leads to responsive dilatation of the proximal arteriole and therefore the glomerular filtration pressure is reduced, leading to a protection of renal glomerulus against hemodynamic stress [84]. A considerable improvement in lipid profile was observed after SGLT-2 administration: decreased triglycerides, decreased LDL-cholesterol, increased HDL-cholesterol, and suppression of generating small oxidized LDL-cholesterol molecules [85].

These data prove that the administration of SGLT-2 inhibitors has protective effects, opposed to almost all the pathophysiological mechanisms that insulin resistance generates in patients with T2DM [84], and serves as a useful therapy in clinical practice.

Numerous studies proved the efficacy of SGLT-2 inhibitors; probably the most cited being EMPAREG-OUTCOME that proved that empagliflozin administration, in T2DM patients and cardiovascular pathology, reduced the cardiovascular mortality by 38% (HR: 0.62; 95% CI: 0.49–0.77; *p* < 0.001) [86]. Also, the hospitalization of T2DM patients for heart failure was reduced by 35% [33]. CANVAS study demonstrated that canagliflozin administration reduced with 14% the incidence of 3Point-Major Advance Cardiovascular Events (3P-MACE) (nonfatal stroke, nonfatal myocardial infarction and cardiovascular death) [34].

ADA 2018 mentions that canagliflozin and empagliflozin (SGLT-2 inhibitors] as well as liraglutide (GLP-1 agonists] significantly reduce cardiovascular risk. The American Association of Endocrinologists recommends GLP-1 agonists as a first choice in initiating dual therapy, followed by SGLT-2 inhibitors [87].

GLP-1 receptor agonists (GLP-1 RA), such as exenatide or lixisenatide, act on post-prandial glycaemia, and as dulaglutide or long-acting release exenatide act on the fasting-glycemia [88]. Both types are efficient in reducing hyperglycaemia; various studies demonstrate that exenatide administrated twice daily in a dosage of 10  μg reduced HbA1c with an average of −0.78% statistically significantly higher than placebo [89]. Long acting GLP-1 RA proved superior to exenatide in improving HbA1c. Exenatide administration (twice a day), had a lower impact than long-acting exenatide administered weekly in DURATION-1 study [90], the first GLP-1 RA reduced HbA1c with −1.5% while the second reduced HbA1c with −1.9% (*p* = 0.0023). Exenatide administered twice a day was also inferior to liraglutide in LEAD-6 study, where liraglutide reduced HbA1c with −1.2% while exenatide reduced Hb1c with −0.79% [91]. GLP-1 RA acts by stimulating glucose-dependent insulin secretion, reducing gastric emptying and increasing satiety, reducing the appetite due to their central action on the hunger centre in the central nervous-system [88].

GLP-1 RA not only reduce hyper-glycemia, helping T2DM to achieve glycaemic targets, but they also have numerous effects on other CVR factors of these patients. GLP-1 RA generally reduce blood pressure; DURATION trials demonstrated a blood pressure reduction between −3 and −5 mmHg with exenatide administration, while in LEAD trials, patients treated with liraglutide benefited from a reduction of systolic blood pressure between −2.7 mmHg and −6.6 mmHg [92,93]. GLP-1 RA also act on blood lipids profile, DURATION studies demonstrating a reduction of total cholesterol between 4.64 and 34.8 mg/dL [94]. Another study revealed that exenatide administered twice-daily reduced LDL-cholesterol with −6% and triglycerides with −12% [95]. The reduction of blood pressure and improvement of lipid profile can be partially attributed to weight loss. Dulaglutide resulted in −1.4 to −3 kg weight loss in AWARD-3 study [96], while in LEAD trials liraglutide administration resulted in weight loss between −1 and −3.2kg. Other pleiotropic effects of GLP-1 RA are improvement of endothelial dysfunction by increasing nitric oxide (NO) production and decreasing the expression of vascular adhesion molecules (VAM) in human endothelial cells [97]. Further, they improve the left ventricle contractility and cardiac output [98] and, in animal models, they help in post-ischemia recovery and increase myocardial viability after ischemic events [99], having natriuretic effects and reducing albuminuria [100]. Receptors for GLP-1 are present in numerous tissues not only in the gut; they are also present in the vascular endothelium, cardiac myocytes, the smooth muscular cells of the arteries but also in the lungs, liver, kidneys, and central nervous system [35]. The LEADER trial, which included 9340 patients with T2DM, demonstrated that liraglutide administration resulted in a 13% reduction of 3-P MACE composite outcome (HR 0.87, 95% CI 0.78–0.97, *p* < 0.001) [35]. In SUSTAIN-6 study, that included 3297 patients with T2DM, administration of semaglutide (in a dose of 0.5 or 1.0 mg) resulted in a statistically significant reduction of 3-P MACE, with 26% (HR 0.74, 95% CI 0.58–0.95]) [101].

In case of T2DM patients with low risk of hypo-glycemia, SGLT2-I and GLP-1RA are efficient alternative therapies and may have positive effects on BP, weight and CV risk. GLP-1 agonists and SGLT-2 inhibitors are superior to current antidiabetic drugs such as sulfonylureas, thiazolidinediones, or DPP-4 inhibitors because of their low risk of hypo-glycemia, their beneficial roles in reducing body weight and reducing the grade of insulin resistance, their action on lowering blood lipids; therefore GLP-1 and SGLT-2 have been promoted as second-line therapeutic agents after metformin [102]. Their values come from their ability in reducing CVR [103] and the fact that therapies such as sulfonylureas, thiazolidinediones, and insulin generate weight gain [104], with all the negative consequences. Moreover, hypo-glycemia caused by sulfonylureas and insulin is associated with a significantly higher CVR because of the arrhythmogenic effect of hypo-glycemia caused by the activation of the sympathetic nervous system [105].

#### 3.2.2. Other Cardiovascular Risk Factors Goals and Management

ADA 2017 and 2018 guides recommend target values of BP under 140/90 mmHg for most diabetic patients and mean values of 130/80 mmHg for patients with high CVR [32,37]. The American Association of Endocrinologists recommends target values of BP under 130/80 mmHg [39,44,74]. The ACCORD BP study has shown that reducing SBP values under 120 mmHg does not offer any additional benefit in comparison to reducing it under 140 mmHg [106]. Multiple classes of anti-hypertensive drugs can be used, although the ideal ones would be ACE inhibitors and ARBs because they reduce the progression of CKD [103,107]. ADA 2017 and 2018 [32,37] guides recommend risk stratification as far as blood fat goals go; patients with atherosclerosis present high-risk respectively those without atherosclerosis present intermediate risk. Patients with high risk should be prescribed high dose statins and those with intermediate risk should be prescribed moderate dose statins, the lipid goals being LDL-cholesterol values of under 70 mg/dL for the former and under 100 mg/dL for the latter. ADA 2018 guide recommends that atherosclerotic patients who do not reach the goal with maximum tolerable statin dose should be added another drug which reduces LDL-cholesterol levels such as ezetimibe or a PCSK9 inhibitor [37]. Aspirin treatment is only recommended for atherosclerotic patients.

Data from multiple guides highlight the fact that the medical therapy should be very carefully chosen in diabetic patients, in such a way that CVR is reduced without any significant side effects. In recent years, there have been anti-diabetic drugs with pleiotropic effects which not only reduce glycaemic values, but also decrease the cardiovascular morbidity and mortality. It is very important to analyse the exact benefit of these drugs through their pleiotropic effect because there are often contraindications for the maximum reduction of the intensity of a CVR factor such as hypertension, thus the effect of the anti-diabetics which offer a cardiovascular benefit can be useful.

## 4. Conclusions

Insulin resistance is a major underlying pathophysiological process that is implied in the occurrence and progression of the major CVR factors in T2DM. As shown in the above analysis, dyslipidaemia, hypertension, being overweight, or obesity and fatty liver disease cluster in patients with T2DM, making them vulnerable for cardiovascular morbidity. Novel risk factors or certainly less explored risk factors such as inflammation, hypercoagulation and endothelial dysfunction can also be associated with insulin resistance and hyperglycaemia. Therapies such as SGLT-2 inhibitors or GLP-1 RA emerge as potent molecules that seem to fight every complication that insulin resistance and hyperglycaemia generate. Their role has been acknowledged in high-quality trails, confirming their capability to reduce the cardiovascular death and morbidity. Therefore, they are not to be neglected in the therapy of T2DM patients.

Before setting objectives as far as glycaemic control, blood pressure control, blood lipids, and thrombosis in diabetic patients, a very careful analysis of the risk/benefit ratio is needed. There are numerous benefits of optimal control such as reducing the risk of microvascular and macrovascular complications; however, side effects can cancel this out due to the risk of hypoglycaemia, weight gain, hypotension, or drug related side effects. Current research data indicate that the metabolic syndrome, insulin resistance, lipid profiles, and diabetes are strongly linked with CVD.
